# Clinical features and risk factors of older adults with bloodstream infection

**DOI:** 10.1186/s12877-025-05934-5

**Published:** 2025-05-31

**Authors:** Mengxi Ding, Yachan Ning, Lipo Song, Xueyuan Yu, Bin Yan, Peijuan Li, Wei Tian, Rongrong Zhang, Wei Chen, Jie Zhen, Fang Lin, Xun Huang, Shuangling Li, Qiao Qin, Zhihong Sun, Yingfeng Wu, Chunmei Wang

**Affiliations:** 1 Department of General Medicine, Xuanwu Hospital, Capital Medical University, Beijing, 100053 China; 2 Department of Intensive Care Medicine, Xuanwu Hospital, Capital Medical University, Beijing, 100053 China; 3 Department of Vascular Surgery, Beijing Luhe Hospital, Capital Medical University, Beijing, 101199 China; 4 Department of General Medicine, West China Hospital, Sichuan University, Chengdu, Sichuan 610044 China; 5 Department of Gerontology, Beijing Jishuitan Hospital, Capital Medical University, Beijing, 100035 China; 6 Department of Intensive Care Medicine, The First Affiliated Hospital of University of Science and Technology of China, Anhui, 230001 China; 7 Department of Intensive Care Medicine, Beijing Shijitan Hospital, Capital Medical University, Beijing, 100038 China; 8 Department of Respiratory, Beijing Friendship Hospital, Capital Medical University, Beijing, 100050 China; 9 Department of Infection, Xiangya Hospital, Central South University, Hunan, 410008 China; 10 Departmeng of Intensive Care Medicine, Peking University First Hospital, Beijing, 100034 China; 11 Department of Pulmonary and Critical Care Medicine of the Second Medical Center, National Clinical Research Center for Geriatric Diseases, Chinese People’s Liberation Army General Hospital, Beijing, 100853 China

**Keywords:** Clinical features, Risk factors, Bloodstream infection, The older adults

## Abstract

**Objective:**

To investigate the clinical features and risk factors of bloodstream infections in the older adults.

**Methods:**

This was a prospective cohort multicenter study. According to the inclusion and exclusion criteria, older adults with suspected bloodstream infections from December 2020 to June 2023 were included in the analysis of the clinical features of older adults bloodstream infections.

**Results:**

A total of 338 older adultswith suspected bloodstream infections were included, of which 141 were diagnosed with bloodstream infections. The proportion of gram-negative bacteria (47.5%) was close to that of gram-positive bacteria (46.1%), while the proportion of gram-negative bacteria was slightly higher.The common clinical manifestations of bloodstream infection were fever (75.2%), decreased level of consciousness (24.8%), chills (14.2%), shock (12.1%), etc.Multivariate regression analysis showed high procalcitonin levels at admission (OR = 2.008, 95%CI 1.258–3.206, *P* = 0.003) and heart rate > 90 beats/min (OR = 2.104, 95%CI 1.302-3.400). *P* = 0.002), arterial partial blood pressure of carbon dioxide < 32 mmHg (OR = 1.922, 95%CI 1.025–3.601, *P* = 0.042), and coronary heart disease (OR = 1.909, 95%CI 1.134–3.213), *P* = 0.015) were independent risk factors for bloodstream infection in the older adults.

**Conclusion:**

The proportion of gram-negative and gram-positive bacteria in bloodstream infections in older adults was similar. The common clinical features of bloodstream infection in the older adults are fever and a decreased level of consciousness, and there was no significant difference in systemic clinical manifestations between bloodstream infection and non-bloodstream infection. High procalcitonin (PCT) level, heart rate > 90 beats/min, arterial partial pressure of carbon dioxide (PaCO2) < 32 mmHg, and underlying coronary heart disease were independent risk factors for bloodstream infection in older adults.

## Background

Bloodstream infection (BSI) refers to the presence of pathogenic microorganisms in a patient’s blood with or without signs and symptoms of infection, including bacteremia and sepsis.As a serious systemic infectious disease, BSI is prone to induce sepsis and multiple organ dysfunction syndrome (MODS) with a high fatality rate, especially in immunosuppressed hosts, and has become a major public health burden worldwide [1]. The older adults are more prone to bloodstream infection due to aging organ function, low immunity, and underlying diseases [2]. One study showed that the average annual incidence of bloodstream infections in patients older than 65 years is more than 6,000 per 100,000 people, and 70% of deaths occur in this age group [3].older adults have more BSI and higher mortality rates. Studies have shown that timely diagnosis and effective anti-infective treatment can significantly reduce the incidence of BSI-related deaths [4]. However, blood culture, which is the gold standard for the clinical diagnosis of BSI, takes a long time and has a low positivity rate. Failure to diagnose BSI early results in missing the best time window for BSI treatment [1, 4]. In recent years, molecular detection techniques have failed to provide immediate diagnosis [1]. Through a prospective multicenter study, this study explored the clinical features and risk factors of bloodstream infection in older adults to identify bloodstream infections in the older adults and provide a scientific basis for clinical prevention and treatment of older adults with bloodstream infections.

## Objects and methods

Prospective multicenter data were collected from 338 older adults with suspected bloodstream infection from December 2020 to June 2023. Inclusion criteria: ①age ≥ 60 years old; ②The group examined included patients who came into the emergency room with suspected sepsis, as well as those already hospitalized; ③No antibiotic use outside the hospital for the first time. Exclusion criteria: ①pregnant women.Diagnostic criteria and procedures: ①When the criteria for suspected bloodstream infection are met, routine microbial culture should be performed before antibiotic treatment, including at least two sets of blood cultures at different sites. If central vein catheters are indwelling, at least one set of blood cultures should be drawn from the central venous catheter. ② Whole blood was examined for aerobic and anaerobic cultures and clinical data were collected.

The National Assessment Report on Aging and Health in China, directed by the World Health Organization and the National Health and Family Planning Commission of China, mentions that the aging population is 60 years old and above.

Relevant diagnostic criteria: BSI diagnostic criteria were defined according to The BSI diagnostic criteria issued by the former Ministry of Health (trial)(No. [2001] 2).

The patient is suspected to have diagnostic criteria for a bloodstream infection: fever and or chills; Systemic inflammatory response syndrome (SIRS) (2 or more criteria); Suspected sepsis with rapid sequential organ failure (qSOFA) score greater than or equal to 2; High levels of C-reactive protein, IL-6 and/or procalcitonin (PCT) and/or 1, 3-beta-D glucan (G test) and/or GM test; The elderly patients may have no fever or low fever during the onset of bacteremia. But there is a decrease in mental state or level of consciousness; Hypotension (systolic blood pressure < 90 mmHg, mean arterial pressure < 70mmHg, or systolic blood pressure drop of more than 40 mmHg, systolic blood pressure below 2 standard deviations of normal age-related values) [5, 6].

This study met medical ethics standards and was approved by the Ethics Committee of our hospital (LYS [2020]103).

Written and oral informed consent from patients or their families was obtained for treatment and testing.

Data collection (1)General patient information: name, age, sex, body mass index (BMI); (2)underlying diseases: hypertension, coronary heart disease, gastroesophageal reflux disease, ischemic cerebrovascular disease, diabetes, chronic renal insufficiency, malignant tumor history with definite pathological diagnosis in the last 5 years; (3) clinical features of bloodstream infection: fever, palpitations, chills, decreased level of consciousness, cough, phlegm, chest tightness, chest pain, nausea, vomiting, abdominal pain, diarrhea, polyuria, urinary urgency, dysuria, dizziness, headache, rash, and shock; (4) complications of bloodstream infection: acute kidney injury, liver injury, abnormal coagulation function, respiratory failure, arrhythmia, gastrointestinal bleeding; (5)①Vital signs: heart rate > 90 beats/min, respiratory rate > 20 beats/min, blood pressure decreased (SBP < 100 or SBP decreased by more than 20%).②Auxiliary examination: Blood culture results were collected when infection was diagnosed and antibiotics were not used.③Laboratory indicators: partial pressure of carbon dioxide in arterial blood (PaCO2), peripheral blood white blood cell count, C-reactive protein, procalcitonin (PCT), and blood lactic acid.(6) The qSOFA score was used to score the cases, and the score result was used as an influencing factor.

Statistical Methods SPSS 27.0 software was used for statistical analysis. Normal distribution data were described as mean ± standard deviation (± s), non-normal distribution data were described as median and quartile, and counting data were represented by examples and percentages (%). The measurement data were compared using the t-test or rank sum test of independent samples, and the counting data were analyzed using the Chi-square test. Variables with *P* < 0.10 in univariate analysis were included in multivariate logistic regression equation analysis (stepwise regression method), and *P* < 0.05 indicated that the difference was statistically significant.

## Results

General condition A total of 338 older adults with suspected bloodstream infection were included, including 228 males (67.5%) and 110 females (32.5%), with an average age of 74.0 (74.03 ± 8.95) years and an average BMI of 23.6 (23.62 ± 3.41) kg/m2. Among 141 patients diagnosed with bloodstream infection, 95 (67.4%) were males and 46 (32.6%) were females. The mean BMI was 23.3 (23.34 ± 3.4) kg/m2, and the mean age was 75.0 (75.01 ± 9.1) years. Among the patients with non-bloodstream infection, there were 133 (67.5%) males and 64 (32.5%) females, with an average BMI of 23.8 (23.81 ± 3.4) kg/m2 and an average age of 73.34 (73.34 ± 8.8) years.There were no significant differences in age, sex, and BMI between the older adults with bloodstream infection and non-bloodstream infection (*P* > 0.05). The case fatality rates of the older adults with BSIand non-BSI were 14.9% and 22.8%, respectively (Table 1).

Clinical Manifestations of 338 patients with suspected bloodstream infections were included in this study. The common clinical manifestations were fever (70.4%), decreased consciousness (26.0%), chills (11.5%), and shock (10.7%). A total of 141 patients were diagnosed with bloodstream infection. The common clinical manifestations were fever (75.2%), decreased consciousness (24.8%), chills (14.2%), and shock (12.1%). There was no significant difference in the systemic clinical manifestations between bloodstream infections and non-bloodstream infections in the older adults, all *P* > 0.05 (Table 1).

Single factor analysis Among the 338 older adults with suspected bloodstream infection, 141 were diagnosed with bloodstream infection. The results of univariate analysis showed that age, high qSOFA score, heart rate > 90 beats/min, PaCO2 < 32 mmHg, and high PCT. There were significant differences in coronary heart disease, coagulation dysfunction, gastroesophageal reflux disease, ischemic cerebrovascular disease, and acute kidney injury between the two groups, all *P* < 0.10 (Table 1).

**Table 1:** Univariate analysis of clinical data of suspected bloodstream infection in multicenter older adults from December 2020 to June 2023

Clinical features		Total (*N* = 338)	Older adults BSI (*N* = 141)	Older adults no BSI(*N* = 197)	*P* value
Gender	Male	228(67.5%)	95(67.4%)	133(67.5%)	0.979
	female	110(32.5%)	46(32.6%)	64(32.5%)	
Age (year)		74.03 ± 8.95	75.01 ± 9.1	73.34 ± 8.8	0.072
BMI (kg/m2)		23.62 ± 3.41	23.34 ± 3.4	23.81 ± 3.4	0.210
Hypertension		187(55.3%)	77(54.6%)	110(55.8%)	0.823
Diabetes		104(30.8%)	49(34.8%)	55(27.9%)	0.180
Coronary heart disease		87(25.7%)	45(31.9%)	42(21.3%)	0.028
Ischemic cerebrovascular disease		61(18.0%)	32(22.7%)	29(14.7%)	0.060
Malignant tumor history with definite pathological diagnosis in recent 5 years		43(12.7%)	18(12.8%)	25(12.7%)	0.984
Gastroesophageal reflux disease		18(5.3%)	11(7.8%)	7(3.6%)	0.086
Chronic renal insufficiency		10(3.0%)	6(4.3%)	4(2.0%)	0.387
Heart rate > 90 beats/min		202(59.8%)	101(71.6%)	101(51.3%)	< 0.001
Respiratory rate > 20 beats/min		160(47.3%)	72(51.1%)	88(44.7%)	0.246
PaCO2 < 32mmHg		53(15.7%)	31(22.0%)	22(11.2%)	0.007
Blood pressure decreased (SBP < 100 or SBP decreased by more than 20%)		33(9.8%)	14(9.9%)	19(9.6%)	0.931
qSOFA score		1.01 ± 1.02	1.18 ± 1.03	0.88 ± 0.99	0.004
High PCT		156(46.2%)	82(58.2%)	74(37.6%)	< 0.001
High C-reactive protein		242(71.6%)	104(73.8%)	138(70.1%)	0.456
High blood lactic acid		75(22.2%)	36(25.5%)	39(19.8%)	0.211
Fever		238(70.4%)	106(75.2%)	132(67.0%)	0.105
Decreased consciousness		88(26.0%)	35(24.8%)	53(26.9%)	0.667
Chill		39(11.5%)	20(14.2%)	19(9.6%)	0.198
Shock		36(10.7%)	17(12.1%)	19(9.6%)	0.478
Palpitations		26(7.7%)	9(6.4%)	17(8.6%)	0.445
Dizziness		8(2.4%)	4(2.8%)	4(2.0%)	0.906
Headache		4(1.2%)	1(0.7%)	3(1.5%)	0.863
Rash		3(0.9%)	2(1.4%)	1(0.5%)	0.770
Phlegm		71(21.0%)	29(20.6%)	42(21.3%)	0.867
Cough		69(20.4%)	31(22.0%)	38(19.3%)	0.544
Chest tightness		37(10.9%)	14(9.9%)	23(11.7%)	0.612
Chest pain		6(1.8%)	1(0.7%)	5(2.5%)	0.402
Vomiting		18(5.3%)	7(5.0%)	11(5.6%)	0.803
Nausea		17(5.0%)	5(3.5%)	12(6.1%)	0.291
Abdominal pain		40(11.8%)	17(12.1%)	23(11.7%)	0.915
Diarrhea		11(3.3%)	6(4.3%)	5(2.5%)	0.571
Polyuria		5(1.5%)	2(1.4%)	3(1.5%)	1.000
Urinary urgency		4(1.2%)	2(1.4%)	2(1.0%)	1.000
Dysuria		2(0.6%)	1(0.7%)	1(0.5%)	1.000
Abnormal coagulation function		51(15.1%)	28(19.9%)	23(11.7%)	0.038
Respiratory failure		50(14.8%)	23(16.3%)	27(13.7%)	0.506
Acute kidney injury		23(6.8%)	14(9.9%)	9(4.6%)	0.054
Liver injury		23(6.8%)	13(9.2%)	10(5.1%)	0.136
Arrhythmia		19(5.6%)	11(7.8%)	8(4.1%)	0.141
Gastrointestinal bleeding		8(2.4%)	5(3.5%)	3(1.5%)	0.399

*High PCT: more than 0.5 ng/ml

Multivariate regression 10 variables (*P* < 0.1)in the univariate analysiswere included in the multivariate logistic regression analysis, and the results showed that heart rate > 90 beats/min, PaCO2 < 32 mmHg, high PCT level, and coronary heart disease were independent risk factors for the diagnosis of bloodstream infection in the older adults (Table 2).

**Table 2:** Multivariate analysis of clinical data of suspected bloodstream infection in multicenter older adults from December 2020 to June 2023

因素	β	S_b_	Waldχ^2^	*P*	OR	95%CI
Heart rate > 90 beats/min	0.744	0.245	9.235	0.002	2.104	1.302-3.400
PaCO2 < 32mmHg	0.653	0.320	4.155	0.042	1.922	1.025–3.601
High PCT	0.697	0.239	8.535	0.003	2.008	1.258–3.206
Coronary heart disease	0.646	0.266	5.920	0.015	1.909	1.134–3.213
Constant	-1.401	0.235	35.592	0.000	0.246	—

Distribution of pathogens Among 141 older adults with bloodstream infections, there were 132 (93.6%) cases of bacterial infection, and the proportion of gram-negative bacteria and gram-positive bacteria was close, 67 (47.5%) were gram-negative bacteria, and 65 (46.1%) were gram-positive bacteria. The common pathogens were Klebsiella pneumoniae, Staphylococcus epidermidis, Escherichia coli, Staphylococcus hominis, and Staphylococcus aureus (Table 3).

**Table 3:** Distribution of pathogenic bacteria of bloodstream infection in multicenter older adults from December 2020 to December 2023

Bacterial distribution	Number and percentage of cases
Ggram-Positive bacteria	65(46.1%)
*Staphylococcus epidermidis*	21(14.89%)
*Staphylococcus hominis*	10(7.09%)
*Staphylococcus aureus*	7(4.96%)
*Enterococcus faecalis*	7(4.96%)
*Enterococcus faecium*	5(3.55%)
*Staphylococcus capitis*	4(2.84%)
*Viridans Streptococci*	1(0.71%)
*Staphylococcus auricularis*	1(0.71%)
*Streptococcus oralis*	1(0.71%)
*Enterococcus avium*	1(0.71%)
*Enterococcus casselifavus*	1(0.71%)
*Staphylococcus caprae*	1(0.71%)
*Streptococcus sanguis*	1(0.71%)
*Streptococcus pharyngobuccalis*	1(0.71%)
*Bacillus licheniformis*	1(0.71%)
*Bacillus subtilis*	1(0.71%)
*Resistance to corynebacterium*	1(0.71%)
Gram-Negative bacteria	67(47.5%)
*Klebsiella pneumoniae*	24(17.02%)
*Escherichia coli*	18(12.77%)
*Pseudomonas aeruginosa*	4(2.84%)
*Baumanii*	3(2.13%)
*Citrobacter fraudiensis*	2(1.42%)
*Stenotrophomonas maltophilia*	2(1.42%)
*Serratia marcescens*	2(1.42%)
*Klebsiella oxytoca*	1(0.71%)
*Bacteroides fragilis*	1(0.71%)
*propionibacterium acnes*	1(0.71%)
*Masai Timone*	1(0.71%)
*Bacteroides polymorphus*	1(0.71%)
*Corynebacterium nonfermentans*	1(0.71%)
*Acinetobacter pieteri*	1(0.71%)
*Salmonella*	1(0.71%)
*Aeromonas villongensis*	1(0.71%)
*Burkholderia cepacia*	1(0.71%)
*Enterobacter cloacae*	1(0.71%)
*Acinetobacter Johnson*	1(0.71%)
**Fungus**	8(5.67%)
*Candida parapsilosis*	5(3.55%)
*Candida glabrata*	2(1.42%)
*Candida albicans*	1(0.71%)
**Mixed infection**	1(0.71%)
*Bifidobacterium longum、Candida glabrata*	1(0.71%)

## Discussion

This prospective multicenter study found that the pathogenic Gram-negative and Gram-positive bacteria were closely related in older adults with BSI. The common clinical manifestations of BSI in the older adults are fever, decreased consciousness, and shock. Compared to non-bloodstream infections in the older adults, the specificity is poor. High PCT level, heart rate > 90 beats/min, PaCO2 < 32 mmHg, and previous coronary heart disease were independent risk factors for bloodstream infection in the older adults.

In this study, 141 older adults with bloodstream infections were included, and the proportion of gram-negative bacteria (47.5%) and gram-positive bacteria (46.1%) was close. The proportion of gram-negative bacteria was slightly higher, and the most common strain was Klebsiella pneumoniae. As in this study, Yaara et al. review found that gram-negative bacteria are common isolates in the older adults (about 40–60% of BSI) [3]. Tang et al. retrospectively analyzed 151 older adults patients diagnosed with BSI, and the distribution of pathogenic bacteria was mainly gram-negative (49.7%). Studies have also shown that K. pneumoniae is the most common cause of BSI in the older adults [7]. In contrast to the present study, one study included patients diagnosed with BSI between 2007 and 2016 and found that the proportion of gram-negative bacteria (70.33%) was much higher than that of gram-positive bacteria (29.67%) [8]; adults ≥ 18 years of age were included in the above studies, and the older adults were included in this study, and it was found that the proportion of gram-positive bacteria increased (46.1%). Wu et al. included patients with BSI treated in an ICU from 2018 to 2020 and found that gram-positive bacteria (44.8%) accounted for a higher proportion than gram-negative bacteria (40.8%), which may be related to the patients included in the ICU in the above studies [9].

The clinical manifestations of bloodstream infections in older adults may not be typical of young people, with no fever or low fever [3]. This study included 141 older adults diagnosed with BSI, and the most common clinical manifestation was fever (75.2%). There were 24.8% older adults with BSI without fever. In addition, older adultswith BSI may have non-specific symptoms of infection such as changes in mental state, decreased appetite, and general malaise [3]. In this study, 24.8% of older adults BSI showed a decreased level of consciousness. The study by Caterino et al., which included 424 patients, found that changes in mental status were common in infected older adults aged 65 years and older. However, changes in consciousness are less than 20% sensitive to determineinfection, possibly because some noninfectious diseases also cause changes in consciousness [10]. Although changes in consciousness are not sufficient as an auxiliary diagnostic basis for infection, Marcantonio emphasized that for new symptoms of delirium, that is, a manifestation of a disturbance of consciousness, the possibility of infectious disease must first be considered to rule out, since infection is one of the most common causes of delirium [11]. The clinical manifestations of BSI in the older adults are not typical, and early identification of BSI in the older adults through clinical manifestations is difficult. Simultaneously, for non-specific signs of infection in older adults, doctors and caregivers should remain highly alert to identify potential bloodstream infection risks.

This study found that high PCT level was an independent risk factor for BSI in the older adults. The traditional PCT in this study further confirmed that a high PCT level is also an independent risk factor for BSI in the older adults. The Chinese Medical Association suggests that infection-related laboratory indicators, including PCT and CRP, should be completed within 3 h for patients with suspected bloodstream infections to determine whether infectious diseases exist [1]. Yang et al. retrospectively analyzed 505 cases of BSI and 102 cases of local bacterial infection and found that when the PCT threshold was greater than 0.675 ng/ml, the AUC for diagnosing BSI reached 0.88, sensitivity was 73.13%, and specificity was 87.25% [4]. A study including 175 patients with BSI found that PCT in the BSI group was significantly higher than that in non-BSI patients, which could be used for the diagnosis of BSI [12].

This study found that older adults with an increased heart rate of > 90 beats/min and previous coronary heart disease were more likely to develop BSI. These results are similar to those reported by Qi et al. Qi et al. analyzed 14,930 patients from the MIME-IV database, including 1444 BSI patients, and established a nomogram prediction model for the early diagnosis of bloodstream infection in the intensive care unit, which was divided into BSI, negative blood culture, and no blood culture groups. It was found that compared with the negative blood culture group, the BSI group was better than the negative blood culture group. There were statistically significant differences in myocardial infarction and heart rate among the underlying diseases (*P* = 0.048 and *p* < 0.001, respectively). There were also statistically significant differences in myocardial infarction and heart rate between the BSI and non-BSI groups (*P* = 0.002 and *P* < 0.001, respectively). After further LASSO regression, the final heart rate was included in the model and assigned. With an increase in the heart rate, its corresponding score would also increase. When the heart rate was 70 beats/h, the score was 10. When the heart rate reached 150 beats/h, the score was 40 points [13].

This study found that PaCO2 < 32 mmHg was an independent risk factor for BSI in old age. Patients with diabetic ketoacidosis, septic shock, renal failure, drug or toxin intake, and loss of gastrointestinal or renal HCO- are represented by metabolic acidosis, which further leads to hyperventilation in patients with deep and rapid breathing aimed at reducing blood carbon dioxide levels to combat metabolic acidosis [14, 15]. In this study, there were 72(51.1%) cases with a respiratory rate > 20 breaths/min in the bloodstream infection group and 88(44.7%) cases in the non-bloodstream infection group, with no significant difference between the two groups (*P* > 0.05). The respiratory rate of the older adults with BSI infection may not be fast, which may be due to the deepening of respiration and increased CO2 emissions. PaCO2 < 32mmHg.

### Advantages and limitations of this study

This study aims to provide help in identifying early the clinical signs of bacteremia in old patients in order to set up early empirical therapy, older adultswhich will have very positive practical significance. In addition, this was a prospective cohort study with multicenter cooperation. However, this study used blood culture to diagnose bloodstream infection, which may lead to some patients with bloodstream infection not being diagnosed because of a low positive rate, and molecular detection technology has a high positive rate. However, the need for genetic diagnostic technology is expensive and difficult to perform in all hospitals, and blood culture remains the gold standard for clinical diagnosis of bloodstream infections.

In summary, this multicenter prospective cohort study showed that the pathogenic bacteria in older adults with bloodstream infections were mainly gram-negative bacteria. The most common clinical manifestation in older adults with BSI was fever, while some patients did not have fever and may present non-specific infection symptoms, such as a decreased level of consciousness. High PCT level, heart rate > 90 beats/min, coronary heart disease, and PaCO2 < 32 mmHg were independent risk factors for blood flow infection in the older adults at admission. The above factors have scientific guiding significance for the early identification of bloodstream infections in older adults, early intervention measures, and improvement of patient prognosis. High-quality, large-sample, randomized controlled trials are needed in the future to further confirm the conclusions of this study.

## Data Availability

The datasets used and analyzed for the current study are available from the corresponding author on reasonable request.

## Abbreviations

BSI: Bloodstream infection
MODS: multiple organ dysfunction syndrome
PaCO2: partial pressure of carbon dioxide in arterial blood
PCT: procalcitonin
qSOFA: quick sequential organ failure assessment
BMI: body mass index
